# Bis(methanol-κ*O*)bis­(quinoline-2-carboxyl­ato-κ^2^ *N*,*O*)nickel(II)

**DOI:** 10.1107/S1600536811041134

**Published:** 2011-10-12

**Authors:** Juhye Kang, Jin Kie Yeo, Pan-Gi Kim, Cheal Kim, Youngmee Kim

**Affiliations:** a Department of Fine Chemistry, Seoul National University of Science and Technology, Seoul 139-743, Republic of Korea; b Department of Forest Genetic Resources, Korea Forest Research Institute, Suwon 441-847, Republic of Korea; c Department of Forest & Environment Resources, Kyungpook National University, Sangju 742-711, Republic of Korea; d Deaprtment of Chemistry and Nano Science, Ewha Womans University, Seoul 120-750, Republic of Korea

## Abstract

In the title complex, [Ni(C_10_H_6_NO_2_)_2_(CH_3_OH)_2_], the Ni^II^ ion lies on an inversion center and is coordinated by two quinoline-2-carboxyl­ate ligands in the equatorial sites and two axial methanol ligands, forming a distorted octa­hedral environment. In the crystal, mol­ecules are linked *via* O—H⋯O hydrogen bonds into a two-dimensional network parallel to (10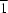).

## Related literature

For inter­actions of metal ions with amino acids, see: Daniele *et al.* (2008 ▶); Parkin (2004 ▶); Tshuva & Lippard (2004 ▶); Stoumpos *et al.* (2009 ▶). For related structures, see: Lee *et al.* (2008 ▶); Park *et al.* (2008 ▶); Shin *et al.* (2009 ▶); Song *et al.* (2009 ▶); Yu *et al.* (2008 ▶, 2009 ▶, 2010 ▶); Kim *et al.* (2011 ▶).
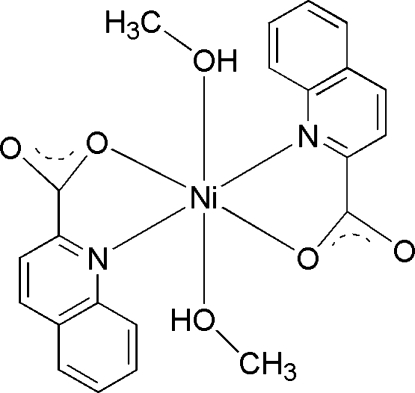


## Experimental

### 

#### Crystal data

[Ni(C_10_H_6_NO_2_)_2_(CH_4_O)_2_]
*M* *_r_* = 467.11
Monoclinic, 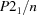

*a* = 10.411 (2) Å
*b* = 7.3910 (15) Å
*c* = 13.556 (3) Å
β = 108.57 (3)°
*V* = 988.8 (3) Å^3^
*Z* = 2
Mo *K*α radiation
μ = 1.03 mm^−1^
*T* = 293 K
0.40 × 0.10 × 0.10 mm

#### Data collection

Bruker SMART CCD area-detector diffractometer
Absorption correction: multi-scan (*SADABS*; Bruker, 1997 ▶) *T*_min_ = 0.884, *T*_max_ = 0.903
5292 measured reflections
1929 independent reflections
1666 reflections with *I* > 2σ(*I*)
*R*_int_ = 0.018

#### Refinement

*R*[*F* ^2^ > 2σ(*F* ^2^)] = 0.027
*wR*(*F* ^2^) = 0.076
*S* = 1.07
1929 reflections
146 parameters
1 restraint
H atoms treated by a mixture of independent and constrained refinement
Δρ_max_ = 0.22 e Å^−3^
Δρ_min_ = −0.31 e Å^−3^

### 

Data collection: *SMART* (Bruker, 1997 ▶); cell refinement: *SAINT* (Bruker, 1997 ▶); data reduction: *SAINT*; program(s) used to solve structure: *SHELXS97* (Sheldrick, 2008 ▶); program(s) used to refine structure: *SHELXL97* (Sheldrick, 2008 ▶); molecular graphics: *SHELXTL* (Sheldrick, 2008 ▶); software used to prepare material for publication: *SHELXTL*.

## Supplementary Material

Crystal structure: contains datablock(s) I, global. DOI: 10.1107/S1600536811041134/lh5343sup1.cif

Structure factors: contains datablock(s) I. DOI: 10.1107/S1600536811041134/lh5343Isup2.hkl

Additional supplementary materials:  crystallographic information; 3D view; checkCIF report

## Figures and Tables

**Table 1:** Hydrogen-bond geometry (Å, °)

*D*—H⋯*A*	*D*—H	H⋯*A*	*D*⋯*A*	*D*—H⋯*A*
O3—H3*O*⋯O2^i^	0.86 (1)	1.81 (1)	2.655 (2)	167 (2)

Symmetry code: (i) 
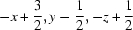
.

**Fig. 1.**
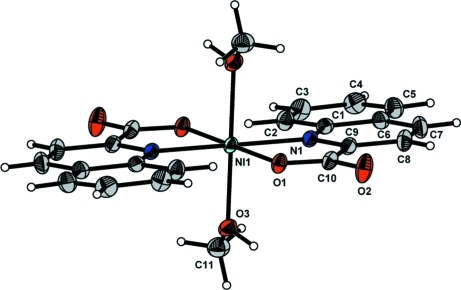
The molecular structure of the title compound with displacement ellipsoids shown at the 30% probability level. Unlabeled atoms are related by the symmetry operator (-x+2, -y, -z+1).

## supplementary crystallographic information

### Comment

The interaction of transition metal ions with biologically active molecules such as amino acids, proteins, sugars, and various acids is of great importance in biological systems (Daniele, *et al.*, 2008; Parkin, 2004; Tshuva & Lippard, 2004; Stoumpos, *et al.*, 2009). As models to examine the interaction, we have intensively studied the interaction of transition metal ions with various acids such as benzoic acid, fulvic acids and humic acids and have reported a variety of structures of copper(II), cadmium(II), and zinc(II) benzoates with quinoxaline,6-methylquinoline, 3-methylquinoline, *trans*-1-(2-pyridyl)-2-(4-pyridyl)ethylene, and di-2-pyridyl ketone (Lee, *et al.*, 2008; Yu,*et al.*, 2008; Park, *et al.*, 2008; Shin, *et al.*, 2009; Song, *et al.*, 2009; Yu,*et al.*, 2008,2009,2010; Kim, *et al.*, 2011). In this work, we have employed nickel(II) chloride as a building block and quinaldic acid as a ligand. We report herin the structure of the title complex.

In the crystal structure of the title compound, [Ni(C_10_H_6_NO_2_)_2_(CH_3_OH)_2_], the Ni^II^ ion occupies a crystallographic inversion center. Two quinoline-2-carboxylate ligands coordinate the Ni^II^ ion in the equatorial sites and two methanol ligands coordinate the Ni^II^ ion in axial sites to form a distorted octahedral environment (Fig. 1). In the crystal, molecules are linked via O—H···O hydrogen bonds to form a two-dimensional network parallel to [1 0 -1].

### Experimental

Quinaldic acid (17.7 mg, 0.1 mmol) and NH_4_OH (13.9 ml, 0.1 mmol) were dissolved in 4 ml methanol and carefully layered with 4 ml methanol solution of nickel(II) chloride hexahydrate (11.9 mg, 0.05 mmol). Suitable crystals of the title compound for X-ray analysis were obtained in two weeks.

### Refinement

H atoms bonded to C atoms were placed in calculated positions with C—H distances of 0.93-0.96Å. They were included in the refinement in a riding-motion approximation with *U*_iso_(H)= 1.2*U*_eq_(C) or *U*_iso_(H) = 1.5*U*_eq_(C_methyl_). The positions of O—H atoms of the methanol ligands were refined with O—H restraints (0.86 Å) and *U*_iso_(H)= 1.2*U*_eq_(O).

### Crystal data

**Table:**

[Ni(C_10_H_6_NO_2_)_2_(CH_4_O)_2_]	*F*(000) = 484
*M**_r_* = 467.11	*D*_x_ = 1.569 Mg m^−^^3^
Monoclinic, *P*2_1_/*n*	Mo *K*α radiation, λ = 0.71073 Å
Hall symbol: -P 2yn	Cell parameters from 3640 reflections
*a* = 10.411 (2) Å	θ = 2.6–28.1°
*b* = 7.3910 (15) Å	µ = 1.03 mm^−^^1^
*c* = 13.556 (3) Å	*T* = 293 K
β = 108.57 (3)°	Rod, colorless
*V* = 988.8 (3) Å^3^	0.40 × 0.10 × 0.10 mm
*Z* = 2	

### Data collection

**Table:**

Bruker SMART CCD area-detector diffractometer	1929 independent reflections
Radiation source: fine-focus sealed tube	1666 reflections with *I* > 2σ(*I*)
graphite	*R*_int_ = 0.018
φ and ω scans	θ_max_ = 26.0°, θ_min_ = 2.2°
Absorption correction: multi-scan (*SADABS*; Bruker, 1997)	*h* = −12→12
*T*_min_ = 0.884, *T*_max_ = 0.903	*k* = −9→9
5292 measured reflections	*l* = −9→16

### Refinement

**Table:**

Refinement on *F*^2^	Primary atom site location: structure-invariant direct methods
Least-squares matrix: full	Secondary atom site location: difference Fourier map
*R*[*F*^2^ > 2σ(*F*^2^)] = 0.027	Hydrogen site location: inferred from neighbouring sites
*wR*(*F*^2^) = 0.076	H atoms treated by a mixture of independent and constrained refinement
*S* = 1.07	*w* = 1/[σ^2^(*F*_o_^2^) + (0.0451*P*)^2^ + 0.2296*P*] where *P* = (*F*_o_^2^ + 2*F*_c_^2^)/3
1929 reflections	(Δ/σ)_max_ = 0.001
146 parameters	Δρ_max_ = 0.22 e Å^−^^3^
1 restraint	Δρ_min_ = −0.31 e Å^−^^3^

### Special details

**Table:**

Geometry. All e.s.d.'s (except the e.s.d. in the dihedral angle between two l.s. planes) are estimated using the full covariance matrix. The cell e.s.d.'s are taken into account individually in the estimation of e.s.d.'s in distances, angles and torsion angles; correlations between e.s.d.'s in cell parameters are only used when they are defined by crystal symmetry. An approximate (isotropic) treatment of cell e.s.d.'s is used for estimating e.s.d.'s involving l.s. planes.
Refinement. Refinement of *F*^2^ against ALL reflections. The weighted *R*-factor *wR* and goodness of fit *S* are based on *F*^2^, conventional *R*-factors *R* are based on *F*, with *F* set to zero for negative *F*^2^. The threshold expression of *F*^2^ > σ(*F*^2^) is used only for calculating *R*-factors(gt) *etc*. and is not relevant to the choice of reflections for refinement. *R*-factors based on *F*^2^ are statistically about twice as large as those based on *F*, and *R*- factors based on ALL data will be even larger.

### Fractional atomic coordinates and isotropic or equivalent isotropic displacement parameters (Å^2^)

**Table:**

	*x*	*y*	*z*	*U*_iso_*/*U*_eq_	
Ni1	1.0000	0.0000	0.5000	0.02463 (12)	
N1	0.79816 (13)	0.10618 (19)	0.48009 (11)	0.0270 (3)	
O1	0.94209 (12)	0.07175 (19)	0.35013 (9)	0.0314 (3)	
O2	0.78467 (15)	0.2090 (2)	0.22280 (11)	0.0530 (4)	
O3	0.91781 (13)	−0.25715 (18)	0.45429 (10)	0.0362 (3)	
H3O	0.8599 (16)	−0.259 (3)	0.3928 (7)	0.043*	
C1	0.72490 (17)	0.1223 (2)	0.54860 (13)	0.0296 (4)	
C2	0.77500 (19)	0.0457 (3)	0.64897 (15)	0.0360 (4)	
H2	0.8589	−0.0116	0.6698	0.043*	
C3	0.7003 (2)	0.0554 (3)	0.71576 (16)	0.0427 (5)	
H3	0.7331	0.0020	0.7811	0.051*	
C4	0.5748 (2)	0.1451 (3)	0.68685 (17)	0.0453 (5)	
H4	0.5257	0.1514	0.7333	0.054*	
C5	0.5250 (2)	0.2221 (3)	0.59184 (17)	0.0429 (5)	
H5	0.4425	0.2826	0.5740	0.051*	
C6	0.59717 (18)	0.2118 (2)	0.51872 (15)	0.0347 (4)	
C7	0.54761 (19)	0.2841 (3)	0.41796 (17)	0.0414 (5)	
H7	0.4653	0.3455	0.3971	0.050*	
C8	0.61990 (19)	0.2645 (3)	0.35068 (15)	0.0385 (4)	
H8	0.5871	0.3101	0.2833	0.046*	
C9	0.74535 (17)	0.1737 (2)	0.38513 (14)	0.0300 (4)	
C10	0.82890 (17)	0.1504 (3)	0.31228 (14)	0.0315 (4)	
C11	0.8677 (2)	−0.3714 (3)	0.51879 (17)	0.0481 (5)	
H11A	0.9424	−0.4196	0.5740	0.072*	
H11B	0.8167	−0.4690	0.4779	0.072*	
H11C	0.8102	−0.3025	0.5478	0.072*	

### Atomic displacement parameters (Å^2^)

**Table:**

	*U*^11^	*U*^22^	*U*^33^	*U*^12^	*U*^13^	*U*^23^
Ni1	0.02223 (18)	0.03058 (19)	0.01837 (18)	0.00163 (12)	0.00265 (12)	−0.00029 (12)
N1	0.0236 (7)	0.0301 (8)	0.0250 (7)	0.0008 (6)	0.0045 (6)	−0.0006 (6)
O1	0.0268 (6)	0.0448 (7)	0.0206 (6)	0.0047 (6)	0.0045 (5)	0.0017 (6)
O2	0.0390 (7)	0.0871 (12)	0.0295 (7)	0.0141 (8)	0.0060 (6)	0.0189 (8)
O3	0.0352 (7)	0.0369 (7)	0.0291 (7)	−0.0050 (6)	0.0000 (5)	−0.0025 (6)
C1	0.0267 (8)	0.0299 (9)	0.0320 (10)	−0.0015 (7)	0.0089 (7)	−0.0048 (7)
C2	0.0307 (9)	0.0458 (11)	0.0317 (10)	0.0052 (8)	0.0105 (8)	−0.0009 (8)
C3	0.0430 (11)	0.0562 (12)	0.0317 (10)	0.0024 (10)	0.0158 (9)	−0.0019 (9)
C4	0.0414 (11)	0.0561 (13)	0.0459 (12)	−0.0013 (10)	0.0246 (10)	−0.0108 (10)
C5	0.0323 (10)	0.0456 (12)	0.0539 (13)	0.0064 (9)	0.0182 (9)	−0.0057 (10)
C6	0.0292 (9)	0.0328 (10)	0.0419 (11)	0.0019 (8)	0.0110 (8)	−0.0035 (8)
C7	0.0288 (9)	0.0418 (11)	0.0511 (12)	0.0112 (8)	0.0094 (9)	0.0063 (9)
C8	0.0304 (9)	0.0429 (11)	0.0366 (10)	0.0061 (8)	0.0027 (8)	0.0114 (9)
C9	0.0249 (8)	0.0317 (9)	0.0302 (9)	−0.0013 (7)	0.0043 (7)	0.0015 (8)
C10	0.0275 (8)	0.0384 (10)	0.0241 (9)	−0.0002 (8)	0.0017 (7)	0.0025 (8)
C11	0.0544 (13)	0.0428 (12)	0.0447 (12)	−0.0066 (10)	0.0124 (10)	0.0000 (10)

### Geometric parameters (Å, °)

**Table:**

Ni1—O1^i^	1.9979 (12)	C3—C4	1.405 (3)
Ni1—O1	1.9980 (12)	C3—H3	0.9300
Ni1—O3^i^	2.0954 (13)	C4—C5	1.351 (3)
Ni1—O3	2.0954 (13)	C4—H4	0.9300
Ni1—N1	2.1779 (14)	C5—C6	1.423 (3)
Ni1—N1^i^	2.1779 (14)	C5—H5	0.9300
N1—C9	1.326 (2)	C6—C7	1.403 (3)
N1—C1	1.382 (2)	C7—C8	1.363 (3)
O1—C10	1.267 (2)	C7—H7	0.9300
O2—C10	1.231 (2)	C8—C9	1.409 (3)
O3—C11	1.429 (3)	C8—H8	0.9300
O3—H3O	0.859 (2)	C9—C10	1.519 (3)
C1—C2	1.411 (3)	C11—H11A	0.9600
C1—C6	1.424 (2)	C11—H11B	0.9600
C2—C3	1.371 (3)	C11—H11C	0.9600
C2—H2	0.9300		
			
O1^i^—Ni1—O1	180.0	C2—C3—H3	119.5
O1^i^—Ni1—O3^i^	88.71 (6)	C4—C3—H3	119.5
O1—Ni1—O3^i^	91.29 (6)	C5—C4—C3	120.31 (19)
O1^i^—Ni1—O3	91.29 (6)	C5—C4—H4	119.8
O1—Ni1—O3	88.71 (6)	C3—C4—H4	119.8
O3^i^—Ni1—O3	180.00 (7)	C4—C5—C6	120.93 (18)
O1^i^—Ni1—N1	100.86 (6)	C4—C5—H5	119.5
O1—Ni1—N1	79.14 (6)	C6—C5—H5	119.5
O3^i^—Ni1—N1	89.82 (5)	C7—C6—C5	123.07 (17)
O3—Ni1—N1	90.18 (5)	C7—C6—C1	118.33 (17)
O1^i^—Ni1—N1^i^	79.14 (6)	C5—C6—C1	118.60 (17)
O1—Ni1—N1^i^	100.86 (6)	C8—C7—C6	120.06 (17)
O3^i^—Ni1—N1^i^	90.18 (5)	C8—C7—H7	120.0
O3—Ni1—N1^i^	89.82 (5)	C6—C7—H7	120.0
N1—Ni1—N1^i^	180.0	C7—C8—C9	118.69 (18)
C9—N1—C1	118.24 (14)	C7—C8—H8	120.7
C9—N1—Ni1	109.96 (11)	C9—C8—H8	120.7
C1—N1—Ni1	131.69 (11)	N1—C9—C8	123.71 (17)
C10—O1—Ni1	118.31 (11)	N1—C9—C10	116.25 (15)
C11—O3—Ni1	123.34 (12)	C8—C9—C10	120.04 (16)
C11—O3—H3O	107.6 (15)	O2—C10—O1	124.64 (18)
Ni1—O3—H3O	113.5 (15)	O2—C10—C9	119.26 (16)
N1—C1—C2	119.98 (15)	O1—C10—C9	116.09 (15)
N1—C1—C6	120.95 (16)	O3—C11—H11A	109.5
C2—C1—C6	119.06 (17)	O3—C11—H11B	109.5
C3—C2—C1	120.16 (18)	H11A—C11—H11B	109.5
C3—C2—H2	119.9	O3—C11—H11C	109.5
C1—C2—H2	119.9	H11A—C11—H11C	109.5
C2—C3—C4	120.9 (2)	H11B—C11—H11C	109.5
			
O1^i^—Ni1—N1—C9	175.65 (12)	C2—C3—C4—C5	0.6 (3)
O1—Ni1—N1—C9	−4.35 (12)	C3—C4—C5—C6	1.0 (3)
O3^i^—Ni1—N1—C9	87.00 (12)	C4—C5—C6—C7	177.7 (2)
O3—Ni1—N1—C9	−93.00 (12)	C4—C5—C6—C1	−1.5 (3)
O1^i^—Ni1—N1—C1	−0.27 (16)	N1—C1—C6—C7	−0.3 (3)
O1—Ni1—N1—C1	179.73 (16)	C2—C1—C6—C7	−178.75 (18)
O3^i^—Ni1—N1—C1	−88.92 (15)	N1—C1—C6—C5	179.02 (16)
O3—Ni1—N1—C1	91.08 (15)	C2—C1—C6—C5	0.5 (3)
O3^i^—Ni1—O1—C10	−85.58 (14)	C5—C6—C7—C8	−177.88 (19)
O3—Ni1—O1—C10	94.42 (14)	C1—C6—C7—C8	1.4 (3)
N1—Ni1—O1—C10	3.98 (13)	C6—C7—C8—C9	−1.1 (3)
N1^i^—Ni1—O1—C10	−176.02 (13)	C1—N1—C9—C8	1.4 (3)
O1^i^—Ni1—O3—C11	27.49 (15)	Ni1—N1—C9—C8	−175.15 (15)
O1—Ni1—O3—C11	−152.51 (15)	C1—N1—C9—C10	−179.23 (14)
N1—Ni1—O3—C11	−73.38 (15)	Ni1—N1—C9—C10	4.23 (18)
N1^i^—Ni1—O3—C11	106.62 (15)	C7—C8—C9—N1	−0.3 (3)
C9—N1—C1—C2	177.39 (17)	C7—C8—C9—C10	−179.64 (18)
Ni1—N1—C1—C2	−7.0 (2)	Ni1—O1—C10—O2	176.36 (16)
C9—N1—C1—C6	−1.1 (2)	Ni1—O1—C10—C9	−2.9 (2)
Ni1—N1—C1—C6	174.56 (12)	N1—C9—C10—O2	179.42 (18)
N1—C1—C2—C3	−177.54 (18)	C8—C9—C10—O2	−1.2 (3)
C6—C1—C2—C3	1.0 (3)	N1—C9—C10—O1	−1.3 (2)
C1—C2—C3—C4	−1.5 (3)	C8—C9—C10—O1	178.12 (17)

Symmetry codes: (i) −*x*+2, −*y*, −*z*+1.

### Hydrogen-bond geometry (Å, °)

**Table:**

*D*—H···*A*	*D*—H	H···*A*	*D*···*A*	*D*—H···*A*
O3—H3O···O2^ii^	0.86 (1)	1.81 (1)	2.655 (2)	167.(2)

Symmetry codes: (ii) −*x*+3/2, *y*−1/2, −*z*+1/2.
